# Combining human and machine intelligence for clinical trial eligibility querying

**DOI:** 10.1093/jamia/ocac051

**Published:** 2022-04-15

**Authors:** Yilu Fang, Betina Idnay, Yingcheng Sun, Hao Liu, Zhehuan Chen, Karen Marder, Hua Xu, Rebecca Schnall, Chunhua Weng

**Affiliations:** Department of Biomedical Informatics, Columbia University, New York, New York, USA; School of Nursing, Columbia University, New York, New York, USA; Department of Neurology, Columbia University, New York, New York, USA; Department of Biomedical Informatics, Columbia University, New York, New York, USA; Department of Biomedical Informatics, Columbia University, New York, New York, USA; Department of Biomedical Informatics, Columbia University, New York, New York, USA; Department of Neurology, Columbia University, New York, New York, USA; School of Biomedical Informatics, The University of Texas Health Science Center at Houston, Houston, Texas, USA; School of Nursing, Columbia University, New York, New York, USA; Heilbrunn Department of Population and Family Health, Mailman School of Public Health, Columbia University, New York, New York, USA; Department of Biomedical Informatics, Columbia University, New York, New York, USA

**Keywords:** human–computer collaboration, cohort identification, eligibility prescreening, informatics

## Abstract

**Objective:**

To combine machine efficiency and human intelligence for converting complex clinical trial eligibility criteria text into cohort queries.

**Materials and Methods:**

Criteria2Query (C2Q) 2.0 was developed to enable real-time user intervention for criteria selection and simplification, parsing error correction, and concept mapping. The accuracy, precision, recall, and F1 score of enhanced modules for negation scope detection, temporal and value normalization were evaluated using a previously curated gold standard, the annotated eligibility criteria of 1010 COVID-19 clinical trials. The usability and usefulness were evaluated by 10 research coordinators in a task-oriented usability evaluation using 5 Alzheimer’s disease trials. Data were collected by user interaction logging, a demographic questionnaire, the Health Information Technology Usability Evaluation Scale (Health-ITUES), and a feature-specific questionnaire.

**Results:**

The accuracies of negation scope detection, temporal and value normalization were 0.924, 0.916, and 0.966, respectively. C2Q 2.0 achieved a moderate usability score (3.84 out of 5) and a high learnability score (4.54 out of 5). On average, 9.9 modifications were made for a clinical study. Experienced researchers made more modifications than novice researchers. The most frequent modification was deletion (5.35 per study). Furthermore, the evaluators favored cohort queries resulting from modifications (score 4.1 out of 5) and the user engagement features (score 4.3 out of 5).

**Discussion and Conclusion:**

Features to engage domain experts and to overcome the limitations in automated machine output are shown to be useful and user-friendly. We concluded that human–computer collaboration is key to improving the adoption and user-friendliness of natural language processing.

## INTRODUCTION

Participant recruitment is one of the biggest barriers to successful clinical trial research, often causing costly delays or early terminations of clinical trials.^1^^,^^2^ A major bottleneck in recruitment is eligibility screening, a process where clinical research staff (eg, clinical research coordinators) reviews patients’ medical history for demographics and clinical conditions, collates and matches the patient data to trial eligibility criteria, and identifies eligible patients.^3^^,^^4^ Electronic eligibility prescreening has been pursued as a potential solution^5–8^ by leveraging natural language processing (NLP) to automate cohort query generation from criteria text and query execution against medical records data. Ni et al^9^^,^^10^ proposed an automated eligibility criteria prescreening approach in emergency medicine and pediatric oncology. Hao et al^11^ constructed the Valx system to extract and structure numeric lab test comparison statements from eligibility criteria. Kang et al^12^ developed the EliIE system for information extraction of criteria text. Criteria2Query (C2Q) 1.0 automated information extraction and query formulation from criteria text.^13^ Tseo et al^14^ extended C2Q 1.0 to develop a clinical trial parser with attention-based conditional random field architecture for entity recognition and word2vec embedding clustering for concept mapping. More recently, deep learning-based named entity recognition models for eligibility criteria and entity normalization models were proposed. Liu et al^15^ trained the Att-BiLSTM model to extract entities from the eligibility criteria of COVID-19 trials. Transformer-based models were also proposed and compared for extracting eligibility concepts.^16–18^ Ji et al^19^ compared BERT, BioBERT, and ClinicalBERT models for biomedical entity normalization. Miftahutdinov et al^20^ presented the DILBERT model for drug and disease concept normalization.

Despite these efforts, manual eligibility prescreening is still the standard practice due to the tremendous complexities in criteria text^21^ and the large gap between machines’ output and users’ need for foundational and context-dependent tasks such as concept mapping^22^ which necessitates human judgment beyond machine automation. A systematic review on NLP systems used for clinical research eligibility prescreening highlighted existing NLP systems’ limitations in understanding language semantics and syntax when translating complex eligibility criteria text or mapping them to patient information from the EHR.^21^^,^^23^ For example, the abbreviation “AD” could represent “Alzheimer’s disease” or “Adrenal adenoma”. The entity “AD” in the criterion “actual treatment with other potential disease modifying drugs of AD” in trial NCT01078168 is hard to be disambiguated automatically and needs human judgment. The model developed by Tseo et al^14^ incorrectly mapped “left main stem stenosis” to the overly general concept “stenosis.” Furthermore, our prior study observed the real-world practice for simplifying eligibility criteria text when translating criteria into cohort queries among clinical researchers.^24^ For instance, explanatory text or measurement concepts without a value threshold cannot be queried and hence are excluded.^24^ Such simplification cannot be automated and still requires human intervention given the state of NLP.

To harness the best of machine automation and human intelligence, we developed C2Q 2.0 to enhance human–computer collaboration to generate more accurate and feasible cohort queries. C2Q 2.0 aims to synergize machine efficiency and human intelligence of domain experts for complex concept recognition, normalization, and criteria simplification. In this study, our contributions include an editable user interface with functions for interactive criteria text parsing result modification, portable cohort SQL query formulation based on the Observational Medical Outcomes Partnership (OMOP) Common Data Model (CDM) version 5, and real-time cohort query execution with result visualization. C2Q 2.0 also brings improved negation scope detection, temporal and value normalization modules upon C2Q 1.0. We report the accuracy of the enhanced modules and the usability and usefulness of C2Q 2.0.

## MATERIALS AND METHODS

### C2Q 2.0 design

**Figure 1.**
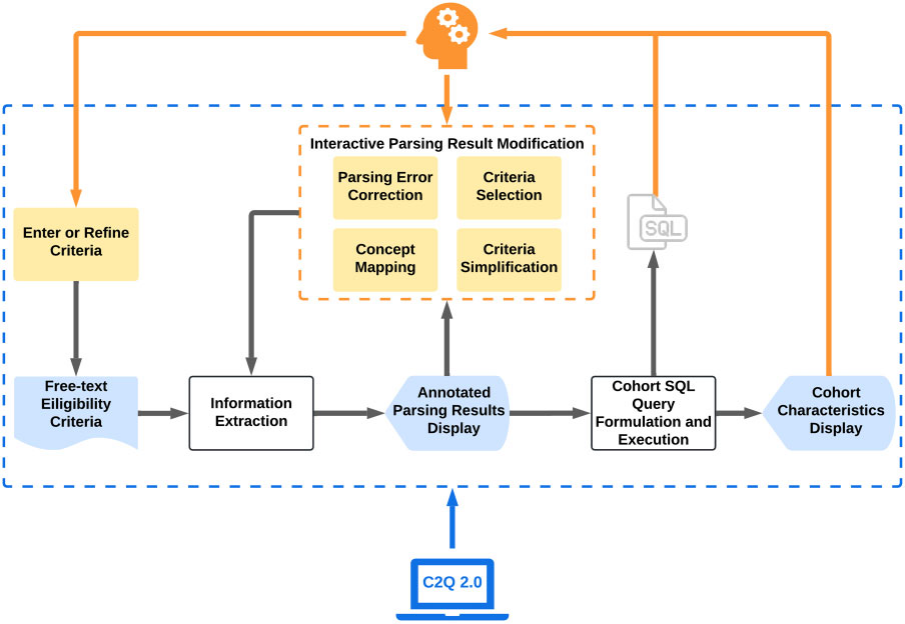
The pipeline of C2Q 2.0.

**Figure 2.**
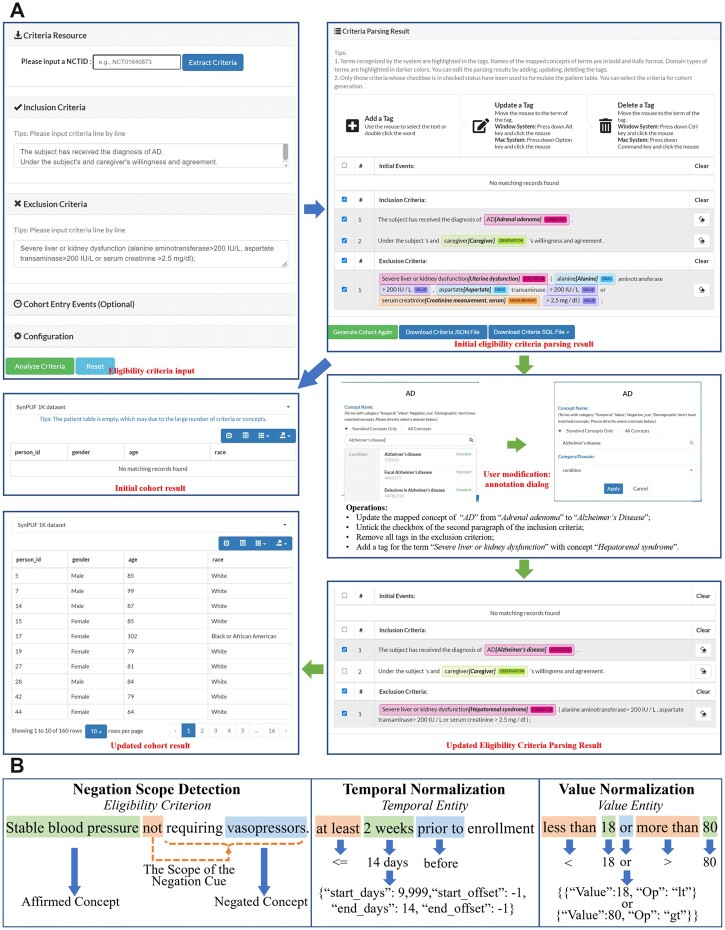
(A) An example of using the editable user interface to generate cohort results after modification. (B) Examples for negation scope detection, temporal and value normalization.

The grey line in Figure 1 represents the machine workflow of C2Q 2.0, and the orange line represents the human intervention. The system first extracts information and generates eligibility criteria representations in JSON format from the free-text eligibility criteria. The recognized entities with their categories and mapped concepts will be displayed to the user, who can review and modify the result as needed. Next, the system continues formulating and executing the cohort SQL queries to identify potentially eligible participants. The user can iteratively modify the criteria and concept mapping as needed to generate new cohorts. The generated cohort’s demographic will be displayed to the user. Figure 2A shows the user interface and an example output for each step.

### Information extraction

After medical entity recognition and concept mapping from the free-text eligibility criteria, C2Q 2.0 detects the scope of negation cues, normalizes the temporal and value entities, and extracts relations, and repeats these steps after user modifications are made. Examples are shown in Figure 2B.

#### Negation scope detection

The negation status of concepts is determined by the scope of the negation cue (eg, “not,” “other than”). We adapted the method for negation scope detection proposed by Khandelwal et al.^25^ We implemented a transfer learning approach based on PubMedBERT,^26^ a pretrained language model from PubMed abstracts and PMC full-text articles, and added a dropout layer with a rate of 0.3 and a classification layer on top it. We adopted the “Augment” preprocessing method for tokenization and the “Average” postprocessing method for combining tokens to get the word-level classification result.^25^^,^^27^ We further turned the word-level results into entity-level classification results based on the frequency of labels “out_of_scope” and “in_scope” among all words in the entity. This negation scope detection model was fine-tuned by the biological abstracts of the Genia corpus and full scientific articles in the BioScope corpus.^28^ It was trained with batch size 8 and Adam optimizer with a learning rate of 3e−5 for 60 epochs. We selected the checkpoint with the highest validation accuracy. After repeating this process 5 times, we selected the one with the highest validation accuracy as our final model.

#### Temporal and value normalization

We normalize temporal entities using “start_days” (ie, the number of days, defined as X), “start_offset” (ie, 1 for “after,” −1 for “before”), “end_days” (defined as Y), and “end_offset” so that an event starts between X days before/after and Y days before/after the index start date. The system recognizes the numbers and their units in a temporal entity by the SUTime library and unifies them to the day unit.^29^ It recognizes the comparison operators in English by the comparison operator’s dictionary and regular expressions and turns them into corresponding symbols (eg, “up to” is converted to “*<=*”). It also recognizes and normalizes the time order words by the same method (eg, “prior to” is converted to “before”). We then turn the free-text temporal attribute into the target CDM format.

C2Q 2.0 normalizes the value entities into value-comparison operator pairs with logical relations. It recognizes the numbers and logic operators (ie, “or,” “and”) by the part-of-speech of each word in a value entity and regular expressions and converts the numbers in word form into digit form using the *NumberNormalizer* class in Stanford JavaNLP.^30^ It identifies and interprets the common mathematical symbols, such as the plus sign “+” and percent sign “%.” It recognizes the comparison operators in the same way as the temporal normalization. Ultimately, it converts the free-text value entity into the target format.

### Interactive criteria parsing result modification

Firstly, users can remove criteria deemed nonqueryable or irrelevant to their prescreening goal. Users can select and add concepts that are not automatically recognized. Users can also simplify complex criteria by removing explanatory text or those extremely general or trivial, nonqueryable, and omittable text. Users can remove wrong concepts extracted from the criteria. Users can modify the concept mapping and categorization results by looking up the concept and category using either fuzzy or exact search supported by ATHENA (https://athena.ohdsi.org). For users who do not know what a standard concept is, the system automatically recommends the standard concepts corresponding to their input.

### Cohort SQL query formulation and execution

We localize and use the SQL query generator and dialect translator (ie, SQL Server and PostgreSQL) from WebAPI in OHDSI to formulate the cohort SQL query formatted by OMOP CDM from the eligibility criteria representation in JSON format. For demonstration purpose, we connected C2Q 2.0 to the publicly available datasets, SynPUF_1K (http://www.ltscomputingllc.com/downloads/) and SynPUF_5% (https://www.ohdsi.org/data-standardization/). They contain a 1000-person sample and a 116 352-person sample of the Centers for Medicare and Medicaid Services (CMS) Linkable 2008–2010 Medicare Data Entrepreneur’s Synthetic Public Use File (DE-SynPUF), respectively. The cohort’s demographic features, including patient ID, age, gender, and race, will be displayed.

### Experimental design

#### Performance of the updated modules

We used a published annotated corpus of the eligibility criteria from 1010 COVID-19 trials for evaluating our methods.^31^ The corpus includes all semantic categories of entities used in C2Q 2.0 and 11 types of relationships. For the evaluation of negation scope detection, we filtered out trials without negation cues in eligibility criteria and retained 308 trials, which included 1223 medical entities in 392 distinct sentences. For the value normalization task, we extracted 2025 distinct value entities from 1010 trials and removed those without digits or other forms of numbers (eg, “positive”), with a wrong text span (eg, “≥” was missed in “≥ 12.0 g/dL”), or cannot be formatted into the CDM structure, such as “two or more doses of > 60 mg or equivalent.” Finally, the test set contained 1603 value entities. For the temporal normalization task, we tested if the time span before or after the reference time point was normalized correctly. We first extracted all 1427 distinct temporal entities from all trials. We dropped those without a clear time span (eg, “currently”), without a specific reference time point (eg, “during the 5-day study”), with a wrong text span (eg, “≥” was missed in “≥4 days before randomization”), with merely a point in time (eg, “on day 0”), or with a too complicated structure (eg, “for ≥ 14 consecutive days in the 4 weeks prior to screening”). The final test set contained 1001 temporal entities. The test set for each evaluation task is displayed in Table 1.

**Table 1.** Test set for each evaluation task

Evaluation task	# of trials	# of entities
Negation scope detection	308	1223
Value normalization	1010	1603
Temporal normalization	1010	1001

For comparison, each task was conducted using C2Q 1.0^13^ and C2Q 2.0. We computed the accuracy, precision, recall, and F1-score for negation scope detection and accuracy for value and temporal normalization. We also implemented the bias-corrected and accelerated (BCa) bootstrap to calculate the 95% confidence interval with 100 000 bootstrap samples for each metric.

### User evaluation

We conducted a usability study to evaluate user perception toward C2Q 2.0’s user engagement features. This study was approved by the Columbia University Irving Medical Center (CUIMC) Institutional Review Board.

#### Protocol selection

We first exported 2257 clinical trials with conditions containing the keyword “Alzheimer” from the Database for Aggregated Analysis of ClinicalTrials.gov (AACT).^32^ Clinical trials without information on the phase, start date, official title, and brief summary were excluded. To conduct a fair and comprehensive evaluation and decrease participant burden, we purposely selected 5 clinical trials on Alzheimer’s disease (AD) with moderately complicated eligibility criteria (ie, approximately 20 to 35 automated recognized entities). Supplementary Table S1 details the 5 selected trials used in the task-based evaluation.

#### Evaluator selection

Evaluators were recruited between July and October 2021 using convenience and snowball sampling.^33^ Recruitment sites included CUIMC, online community forums (eg, Association of Clinical Research Professionals), and collaborators in other academic institutions. To be included, the evaluator must be a current clinical research coordinator. The prescreening survey included a question on AD research experience to ensure the representativeness of evaluators. Evaluations were considered complete when evaluation tasks were done and the valid data in the follow-up surveys were at least 80%. We aimed for 10 complete evaluations.^34^ Those who completed the evaluation were given a $40 gift card as a token of appreciation.

#### Data collection procedures

An email with 2 randomly assigned National Clinical Trial identification numbers (NCTID) and a link to an evaluation survey using the Qualtrics platform (Qualtrics, Provo, UT) was sent to each evaluator. Following the completion of informed consent, evaluators watched a tutorial and demonstration video of C2Q 2.0 before performing the evaluation. C2Q 2.0 first generated the criteria parsing result automatically for each testing trial, and then evaluators could modify this result as they deemed necessary. After the completion of using both NCTIDs successively, evaluators were invited to complete a demographic questionnaire, the Health-ITUES,^35^^,^^36^ and a feature-specific usability questionnaire, respectively. Evaluators completed a demographic questionnaire with information on the evaluators’ clinical research background and experience in eligibility prescreening. The validated Health-ITUES is a customizable questionnaire to measure the usability of a health information technology system.^35^^,^^36^ The questionnaire consists of 20 items rated on a 5-point Likert scale, where a higher scale value indicates higher perceived usability of the system and a cutoff score of 4.3 for high usability.^37^^,^^38^ For this study, 17 Health-ITUES items were modified to address the specific user tasks (Supplementary Table S2). Three out of 4 factors were included: (1) perceived usefulness (Cronbach’s α = .94); (2) perceived ease of use (Cronbach’s α = .95); and (3) user control (Cronbach’s α = .81).^36^ Perceived usefulness evaluates the users’ task accomplishment by using the system. Perceived ease of use and user control evaluate how users interact with the system. The “quality of work life” subscale (3 items) was not included because C2Q was not integrated into the evaluators’ workflow. The overall Health-ITUES score was generated from the mean of the scores for all the items. We conducted the feature-specific evaluation (Supplementary Table S3). Adapted from the USE questionnaire,^39^ we evaluated the user satisfaction and ease of learning of the modification functions. We also collected quantitative data via interaction logging.

## RESULTS

### Performance of the enhanced modules

Table 2 shows that negation scope detection, value, and temporal normalization all greatly improved. Among 1223 entities for the evaluation of negation scope detection, C2Q 1.0 incorrectly identified the negation status of 274 entities, of which 85.8% were correctly identified by C2Q 2.0. For example, in the sentence “If symptomatic, presence of mild to moderate symptoms without signs of respiratory distress, with positive for SARS-CoV-2 diagnostic assay within 72 hours prior to informed consent.”, the negation scope of the negation cue “without” was correctly recognized to include “respiratory distress” but not “symptomatic,” “symptoms,” and “SARS-CoV-2 diagnostic assay.”

**Table 2.** Performance of negation scope detection, value normalization, and temporal normalization modules in C2Q 1.0 and C2Q 2.0 with 95% confidence intervals using COVID-19 trials

Evaluation task	Metric	C2Q 1.0	C2Q 2.0
Negation scope detection	Accuracy	0.776 [0.751, 0.798]	0.924 [0.907, 0.937]
	Precision	0.792 [0.758, 0.823]	0.963 [0.945, 0.977]
	Recall	0.759 [0.724, 0.791]	0.884 [0.857, 0.908]
	F1-score	0.775 [0.748, 0.800]	0.922 [0.905, 0.937]
Value normalization	Accuracy	0.601 [0.576, 0.624]	0.966 [0.955, 0.973]
Temporal normalization	Accuracy	0.554 [0.522, 0.584]	0.916 [0.896, 0.931]

Among 1603 value entities for evaluating value normalization, C2Q 1.0 incorrectly normalized 639 entities, of which 92.6% were correctly converted into the standard format using C2Q 2.0. For example, “34.4-44.6%” was normalized as “Value between 0.344 and 0.446.” Another example is “III or higher,” which was normalized as “Value ≥ 3.”

Among 1001 temporal entities for evaluating temporal normalization, C2Q 1.0 incorrectly normalized 446 entities, of which 83.9% were with a correct normalization using C2Q 2.0. For instance, “at least 5 days before the leukapheresis procedure” was correctly translated as “The event starts between 9999 days before and 5 days before the index start date.” Besides, “Two weeks to 1-year post hospital discharge” was precisely normalized as “The event starts between 14 days after and 365 days after the index start date.”

### Descriptive analysis

We conducted 19 user evaluations but included only 10 in the analysis; the rest were excluded because (1) 2 evaluations appeared to be from the same evaluators because the same cookie was used and the same protocols were evaluated and (2) 7 evaluators did not complete the tasks. The majority of evaluators worked in clinical research for at least a year (80%), had AD clinical research experience (80%), and were involved in prescreening potential participants for research (50%). Table 3 provides details about them.

**Table 3.** Evaluators’ clinical research background^a^

Characteristic	Category	Ten (*n* = 10)	Eight (*n* = 8)
		Included evaluators (%)	Excluded evaluators (%)
Number of years working in clinical research	Less than 1 year	2 (20)	2 (25)
	1 year to less than 5 years	5 (50)	2 (25)
	5 years or over	3 (30)	4 (50)
Alzheimer’s disease clinical research experience	No experience	2 (20)	4 (50)
	Less than 1 year	5 (50)	0 (0)
	1 year or more	3 (30)	4 (50)
Involvement in prescreening potential participants for research	No	5 (50)	1 (12.5)
	Yes	5 (50)	7 (87.5)

There were 18 evaluators in total, 10 of which were included.

**Figure 3.**
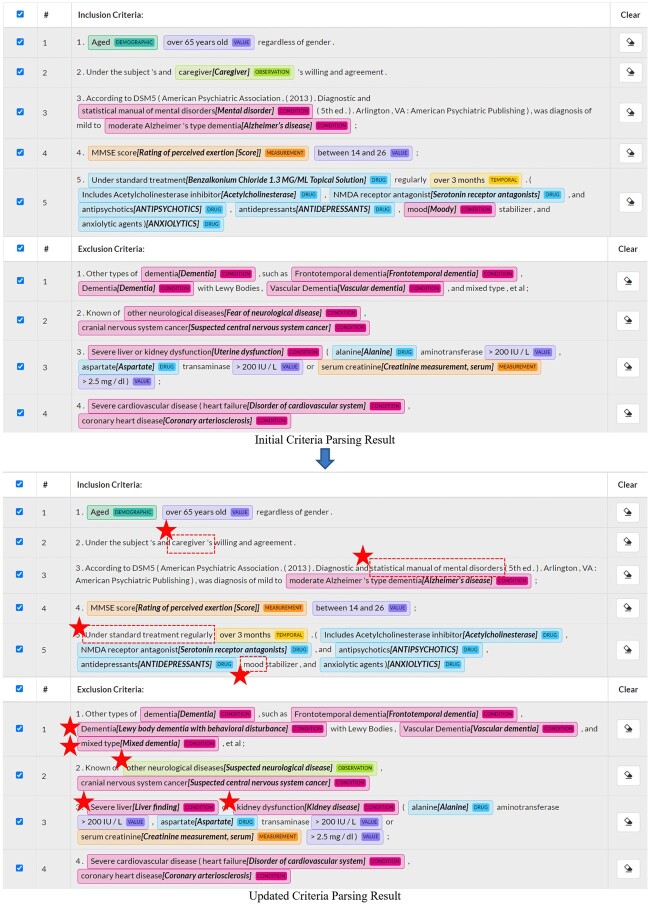
Comparison of the criteria parsing result before and after modifications made by one of the evaluators for the clinical trial NCT04249869. The evaluator is with prescreening involvement and has 1 to 2 years of experience in clinical research and in AD research. The modified medical concepts are highlighted with star icons. The automatically extracted concepts removed by the evaluator are enclosed by dotted line boxes. AD: Alzheimer’s disease.

Figure 3 contrasts the automatically generated criteria parsing result for study NCT04249869 and the updated criteria parsing result after modifications made by one of the evaluators. The modified medical concepts are emphasized with star icons. Using C2Q 2.0, most of the medical concepts were correctly recognized and normalized by machine, but still, some concepts extracted and normalized from the criteria that are nonqueryable were removed by the evaluator, such as “Caregiver.” “Under standard treatment” recognized correctly by machine is too broad so that the evaluator removed it from the query. Some concepts that could not be recognized or correctly normalized by machine were added or updated by the evaluator. For example, the evaluator added the concept “mixed dementia” for the unrecognized entity “mixed type” to the query. The evaluator was able to identify the correct standard OMOP concept for this text and added it to the query. For “Severe liver and kidney dysfunction,” the evaluator broke it down into 2 concepts and added “liver finding” and “kidney disease” to the query.

**Figure 4.**
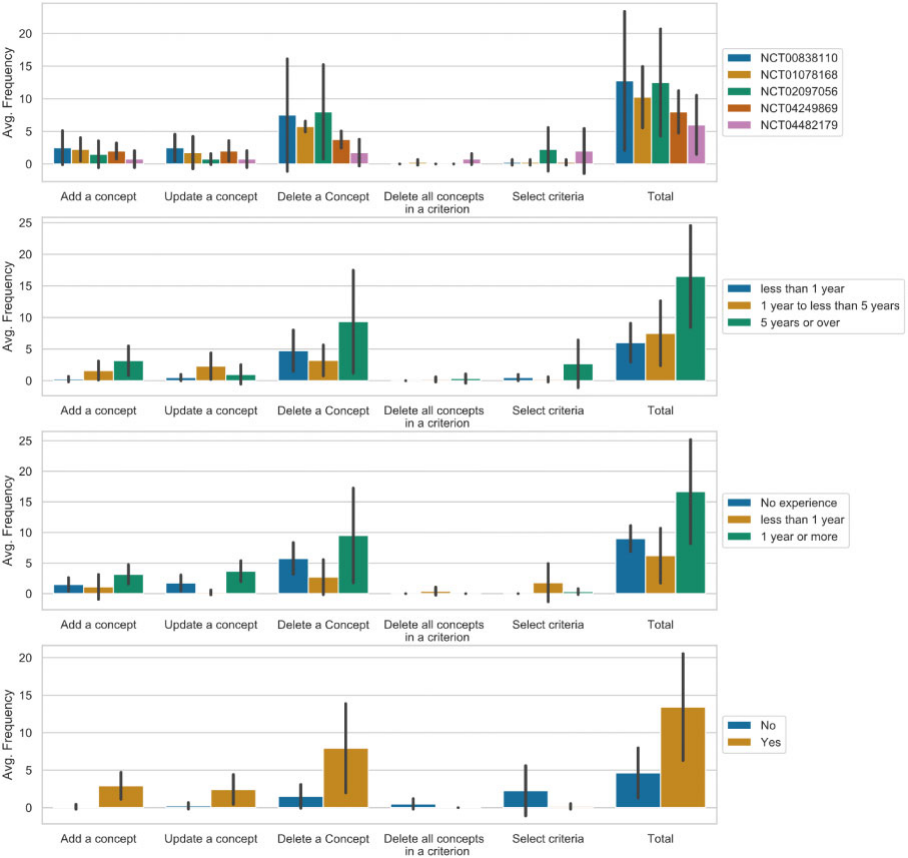
Average frequency of using each function for criteria parsing result’s modification.

On average, the eligibility criteria parsing result received 9.9 modifications per clinical trial. Concept deletion had the highest frequency among all modification functions, including adding a concept (1.80, SD = 2.02), updating a concept (1.55, SD = 1.93), deleting a concept (5.35, SD = 5.82), deleting all concepts in an eligibility criterion (0.2, SD = 0.52), and selecting eligibility criteria (1.00, SD = 2.43). Figure 4 displays the usage of each function stratified by NCTID, research experience length, and prescreening involvement experience. Generally, fewer modifications (6 modifications on average) were made for trial NCT04482179. All evaluators to whom this trial was randomly assigned were with less than 6-month research experience in AD, and 3 out of 4 of them were with no prescreening experience. Besides, evaluators with a longer clinical research experience, at least 1 year of research experience in AD, or prescreening experience, made more modifications. Furthermore, evaluators without trial prescreening experience often deleted automatically generated parsing results and reselected eligibility criteria.

### Usability evaluation

The Health-ITUES results in Table 4 revealed an overall score of 3.84, reflecting moderate usability. Supplementary Figure S1 demonstrates the comparison of the Health-ITUES scores across different groups. Overall usability was the highest (mean = 4.14) in evaluators with 5 years or more clinical research experience. Evaluators involved in prescreening potential participants rated the overall usability lower. Further, evaluators with experience in AD research rated usability higher compared to those without experience. As shown in Table 4, the average learnability of all modification functions was 4.54. Evaluators were satisfied with the automatically generated criteria parsing result (mean = 4.00), the availability of all user engagement features (mean = 4.30), and the parsing result after all modifications (mean = 4.10).

**Table 4.** Health-ITUES and feature-specific usability scores

	Mean (SD)
Health-ITUES	
Perceived usefulness	3.99 (0.66)
Perceived ease of use	3.80 (1.06)
User control	3.73 (0.89)
Overall score	3.84 (0.71)
Feature-specific	
Pleasant to use	3.90 (0.57)
User satisfaction: automatically generated criteria parsing result	4.00 (0.67)
User satisfaction: modified criteria parsing result	4.10 (0.74)
User satisfaction: concept searching	3.60 (0.97)
User satisfaction: annotation dialog	4.20 (0.63)
Easy to learn: add a concept	4.50 (0.53)
Easy to learn: update a concept	4.70 (0.49)
Easy to learn: delete a concept	4.60 (0.52)
Easy to learn: delete all concepts in an eligibility criterion	4.60 (0.52)
Easy to learn: select eligibility criteria	4.30 (0.95)
Availability of all user engagement features	4.30 (0.95)

**Figure 5.**
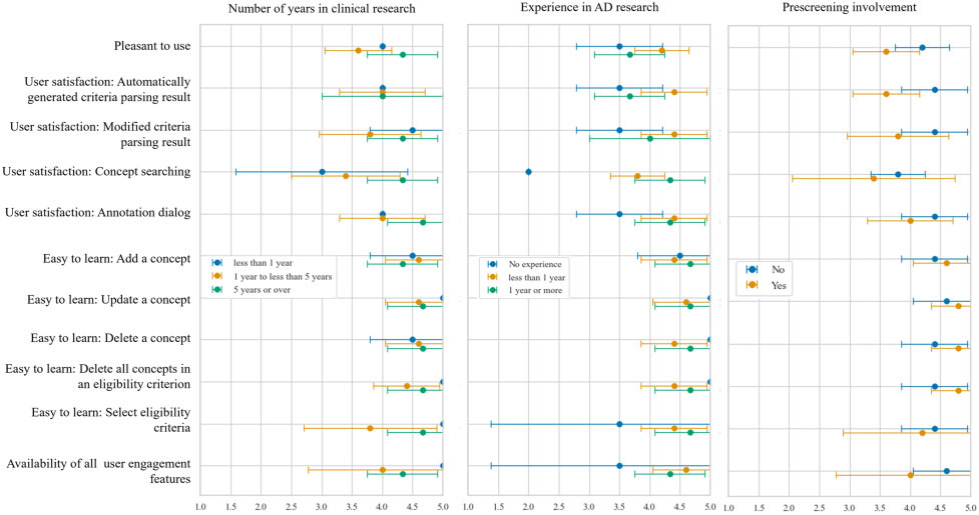
Score comparison of feature-specific usability scores across groups.

Figure 5 shows different feature-specific usability ratings between experienced and less experienced users. The most diverging user satisfaction scores for concept searching function were observed among evaluators with <1 year of clinical research experience. Evaluators with AD research experience were more satisfied with the concept searching function and the interface than those without. Evaluators with prescreening involvement were less satisfied with user engagement features.

We also collected open-ended feedback. Two evaluators expressed that the interface was “straightforward” and “easy to understand and use.” Recommendations were provided for improving flexibility in editing the eligibility criteria text in the criteria parsing result that “allow to edit the automated inclusion and exclusion criteria to add a new criterion.” One evaluator commented, “This is a wonderful tool that is very effective at extracting and mapping criteria from clinical trials.” Furthermore, since automated concept mapping is error-prone (eg, “major medical disorder” was mistakenly mapped to concept “mental disorder”), one evaluator stated, “I wanted to exclude whole paragraphs from the parsing where I was pessimistic that the parsing could actually capture what the intention behind the criterion was.” Two evaluators recommended improving the ease of use by “instead of using keys to edit maybe have dropdowns” and “adding a bit more instructions would be useful.”

## DISCUSSION

C2Q 2.0 combines machine and human intelligence for transforming free-text clinical research eligibility criteria into executable cohort queries. It incorporates the domain expertise of clinical researchers into the process of recognizing and normalizing entities from the eligibility criteria and achieves promising usability. On average, 9.9 human interventions were made to a clinical study. The high frequency of concept deletion is likely due to a great number of medical concepts that are vague or broad (eg, “multimorbidity” in trial NCT01078168, or “major structural brain disease” in trial NCT00838110), unimportant at certain phases (eg, “Alanine,” “Aspartate,” and “Creatinine measurement, serum” in “Severe liver or kidney dysfunction (alanine aminotransferase > 200 IU/L, aspartate transaminase > 200 IU/L or serum creatinine >2.5 mg/dL);” in trial NCT04249869), or not informative (eg, “history of” since diagnoses in the EHR are past history). Human intelligence is needed to tease out and simplify the complex text, as we previously reported.^24^ Another reason for manual deletions is that evaluators wanted to correct the span of the entity to which the concept is mapped. For example, for the phrase “mood stabilizer” in trial NCT04249869, only “mood” was automatically recognized as an entity and mapped to a wrong concept, and one of the evaluators deleted the machine output and added the concept “Mood Stabilizer” to the query. Experienced researchers tend to make more deletion and modify concept mapping since they have a background in the disease of interest and hence are more familiar with the practical criteria for screening. Evaluators without prescreening experience tend to simplify the results and avoid identifying standard concepts for these criteria, partially because they lack knowledge of the nuances involved in eligibility prescreening and which criteria are feasible to query in the EHR.^4^

When comparing the modifications made by different evaluators, we observed variances in user choices. For example, in “contraindication to acitretin such as osteoporosis, hypoalbuminaemia” in trial NCT01078168, concepts “Medical contraindication,” “acitretin,” “Osteoporosis,” and “Hypoalbuminemia” were automatically extracted. Some evaluators removed the “Osteoporosis” and “Hypoalbuminemia” from the query since they are too specific, while others retained both. C2Q 2.0 is flexible to accommodate such individualized customization of criteria queries for different contexts.

The evaluators, especially those without disease-specific domain expertise, were least satisfied with the concept searching function since they lack the domain knowledge necessary for correctly mapping concepts in the eligibility criteria.^40^ Evaluators experienced in clinical research and those with disease-specific domain expertise indicated higher satisfaction. Domain knowledge is a key differentiating factor that influences user experience. Though all evaluators rated the learnability positively, those with prescreening experience found the functions easier to learn and expected more user engagement features, which may be because of the inherent complexity of eligibility criteria (eg, temporality, underspecified requirements).^41^ This finding also implies that the tool’s intended adopters should be equipped with knowledge of clinical concepts and skills for mapping criteria concepts into standard clinical terminologies.

Furthermore, though the user interface exhibited promising usability, the evaluation revealed a margin of improvement, especially in user control during the interface interaction (ie, error messages, recovering from errors, and clear information). This is consistent with the findings of Zheng et al’s^42^ evaluation on the ease of adoption evaluation of 5 clinical NLP systems that showed one of the reasons for unsatisfactory rating is the system’s failure to communicate adequately how the systems work for intended adopters. Interestingly, evaluators involved in prescreening for potentially eligible participants rated the usability lower than those not involved. This may be because those involved in prescreening already have a set workflow that may not be congruent to how the interface works. More in-depth usability evaluations of C2Q 2.0 are warranted and underway.^43^

Finally, on top of the interactive and editable user interface of C2Q 2.0, we can further extend our current system and construct human-in-the-loop machine learning models for named entity recognition, concept mapping, and criteria simplification. They can be improved by iterative fine-tuning with user feedback on the criteria parsing results. This is part of our future research plan. The results of this study confirm the needs and value of incorporating user input to improve accuracy and efficiency for querying.

### Error analysis

The negation scope detection errors were mainly due to the lack of the period sign (“.”) at the end of the eligibility criteria text. For example, the negation scope of the negation cue “except” in the criterion “Known history of autoimmune disease except prior thyroiditis” failed to include the entity “thyroiditis.” However, if we manually added a period to the end of the sentence, it would be successfully detected as negated. One possible explanation is that the model was fine-tuned on a corpus where sentences are complete. Therefore, in the future, we can automatically add a period to the incomplete sentences before detection or retrain the model with mixed data where half of the sentences are erased with their punctuations.

The value normalization errors can be mostly attributed to the units in a value entity. For instance, in the entity “>3 g/24-hours”, not only “3” but also “24” in the unit are normalized as values. Further work is required to detect the unit in value normalization. The temporal normalization errors were largely due to the failure to transform temporal entity in date format (eg, “since January 1, 2020”) to the CDM format. The unnormal format of the unit (eg, “>72 h”), where the numbers cannot be recognized as temporal entities, also leads to the occurrence of errors.

### Limitations of the present study

The accuracy evaluation for negation scope detection, temporal and value expression extraction greatly hinges on the accuracy of the previously annotated COVID-19 clinical trials. Additional studies are warranted to test the generalizability of the results to other clinical trials beyond the COVID-19 studies. Our sample size for usability evaluation was kept at 10, which was sufficient for discovering 80% of usability problems,^34^ but inadequate to investigate how significant the difference is among different user groups. Future studies are warranted to investigate how the findings may generalize to all clinical research coordinators as well as to other clinical research staff (eg, research assistants, research nurses). Besides, although we provided a tutorial video and an instruction for the usage of C2Q 2.0, we have not scientifically confirmed the sufficiency of user training for our evaluators in this study. It is possible that more changes will further improve user satisfaction and user experience. Finally, the protocols used in the study are focused on a specific disease; hence whether the usability testing result is generalizable to other disease-specific studies needs further investigation.

## CONCLUSION

This article reports the design and pilot evaluation of C2Q 2.0, a novel user interface that engages domain experts to semiautomatically generate cohort definition queries from free-text eligibility criteria. The system shows notable improvement in negation scope detection and temporal and value normalization. The descriptive analysis and user evaluation results demonstrate the necessity of user engagement and the flexibility and usability of C2Q 2.0. Domain expertise is instrumental in the full utilization of the system. We conclude that human–computer collaboration promises to enhance NLP.

## FUNDING

Research reported in this publication was supported by the National Library of Medicine grant 5R01LM009886-11, the National Center for Advancing Translational Sciences grants UL1TR001873 and 1OT2TR003434-01, the National Institute of Nursing Research grants T32NR007969 and K24NR018621, and the Agency for Healthcare Research and Quality grant 1R36HS028752. The content is solely the responsibility of the authors and does not necessarily represent the official views of the National Institutes of Health.

## AUTHOR CONTRIBUTIONS

The authors contributed to the study as follows: YF: Project administration, conceptualization, methodology, software, data curation, formal analysis, investigation, validation, visualization, and writing—original draft. BI: Project administration, formal analysis, investigation, validation, visualization, and writing—original draft. YS: Conceptualization, methodology, and writing—reviewing and editing. HL and ZC: Methodology and writing—reviewing and editing. KM and HX: Writing—reviewing and editing. RS: Validation, research supervision, and writing—reviewing and editing. CW: Idea initialization, project administration, conceptualization, methodology, validation, team creation, research supervision, funding acquisition, and writing—reviewing and editing.

## SUPPLEMENTARY MATERIAL

Supplementary material is available at *Journal of the American Medical Informatics Association* online.

## CONFLICT OF INTEREST STATEMENT

Dr. Xu and The University of Texas Health Science Center at Houston have financial conflict interests in Melax Technologies Inc.

## DATA AVAILABILITY

The data underlying this article are available in the article and in its online supplementary material.

## Supplementary Material

ocac051_Supplementary_Data
