# Supplementary material for: Combining human and machine intelligence for clinical trial eligibility querying
Source: J Am Med Inform Assoc. 2022 Apr 15;29(7):1161–71. doi: 10.1093/jamia/ocac051 (PMC9196697; doi:10.1093/jamia/ocac051)

**Supplements**

**Supplementary Table 1.** Clinical Trial Protocols Characteristics.

| **NCTID** | **Phase** | **Count of Automatically Extracted Medical Terms** | | | | | | | | | |
| --- | --- | --- | --- | --- | --- | --- | --- | --- | --- | --- | --- |
|  |  | **Demographic** | **Condition** | **Observation** | **Procedure** | **Drug** | **Measurement** | **Value** | **Temporal** | **Negation Cue** | **Total** |
| NCT00838110 | 3 | 0 | 12 | 3 | 1 | 8 | 1 | 1 | 4 | 1 | 31 |
| NCT01078168 | 2 | 0 | 13 | 2 | 1 | 1 | 2 | 1 | 0 | 0 | 20 |
| NCT02097056 | 4 | 2 | 9 | 3 | 0 | 8 | 2 | 3 | 5 | 2 | 34 |
| NCT04249869 | 1/2 | 1 | 12 | 1 | 0 | 8 | 2 | 5 | 1 | 0 | 30 |
| NCT04482179 | 1 | 0 | 14 | 4 | 2 | 0 | 2 | 1 | 1 | 0 | 24 |

Note: NCTID = National Clinical Trial identification number

**Supplementary Table 2.** Modified Health ITUES.

| Strongly Agree 5 4 3 2 1 Strongly disagree | | |  |
| --- | --- | --- | --- |
| Perceived Usefulness | |  |  |
| 1 | Using Criteria2Query makes it easier to query clinical databases to prescreen for eligible research participants. | | |
| 2 | Using Criteria2Query enables me to generate a query to identify potentially eligible research participants more quickly. | | |
| 3 | Using Criteria2Query makes it more likely that I correctly identify eligible research participants. | | |
| 4 | Using Criteria2Query is useful for prescreening eligible research participants. | | |
| 5 | I think Criteria2Query presents a more equitable process for generating a query to identify potentially eligible research participants. | | |
| 6 | I am satisfied with Criteria2Query for generating a query to identify potentially eligible research participants. | | |
| 7 | I can generate a query to identify potentially eligible research participants because of Criteria2Query. | | |
| 8 | Using Criteria2Query increases my ability to generate a query to identify potentially eligible research participants. | | |
| 9 | I am able to generate a query to identify potentially eligible research participants when I use Criteria2Query. | | |
| Perceived Ease of Use | |  | |
| 10 | I am comfortable with my ability to use Criteria2Query. | | |
| 11 | Learning to operate Criteria2Query is easy for me. | | |
| 12 | It is easy for me to become skillful at using Criteria2Query. | | |
| 13 | I find Criteria2Query easy to use. | | |
| 14 | I can always remember how to log on to and use Criteria2Query. | | |
| User Control | |  | |
| 15 | Criteria2Query gives error messages that clearly tell me how to fix problems. | | |
| 16 | Whenever I make a mistake using Criteria2Query, I recover easily and quickly. | | |
| 17 | The information (such as instructions on how to modify the tagged terms) provided with Criteria2Query is clear. | | |

**Supplementary Table 3.** Feature-specific questionnaire.

| 1 | I am satisfied with the automated criteria parsing result generated by Criteria2Query (the criteria before any modifications were made). | Strongly Agree 5 4 3 2 1 Strongly disagree |
| --- | --- | --- |
| 2 | Did you edit the criteria parsing result in either of the clinical research protocol assigned to you? | Yes/No |
| 3 | What is/are your most common reason/s for modifying the automated parsing result? (Select all that apply) | To change the mapped concept of the term  To change the category of the term  To include a term  To exclude a term  To include an entire paragraph of criterion  To exclude an entire paragraph of criterion  Other |
| 4 | It is pleasant to use the editable user interface to modify the criteria parsing result. | Strongly Agree 5 4 3 2 1 Strongly disagree |
| 5 | The final modified criteria parsing result expresses the appropriate requirements for eligibility prescreening for potential research participants. |  |
| 6 | It was easy to find the correct mapped concept using the concept searching function. |  |
| 7 | I am satisfied with the design of the annotation dialog. |  |
| 8 | It was easy to learn to use the function of adding an annotation/tag for a medical term. |  |
| 9 | It was easy to learn to use the function of updating an annotation/tag for a medical term. |  |
| 10 | It was easy to learn to use the function of removing an annotation/tag for a medical term. |  |
| 11 | It was easy to learn to use the function of removing all annotations/tags in a paragraph. |  |
| 12 | It was easy to learn to use the function of excluding or including an entire paragraph of eligibility criteria. |  |
| 13 | The system provide all the functionality that I need to edit the criteria parsing result. |  |
| 14 | Do you have any feedback on the strengths or areas for improvement for the interactive user interface? | Text entry |

**Supplementary Figure 1.** Comparison of Health-ITUES score across groups.


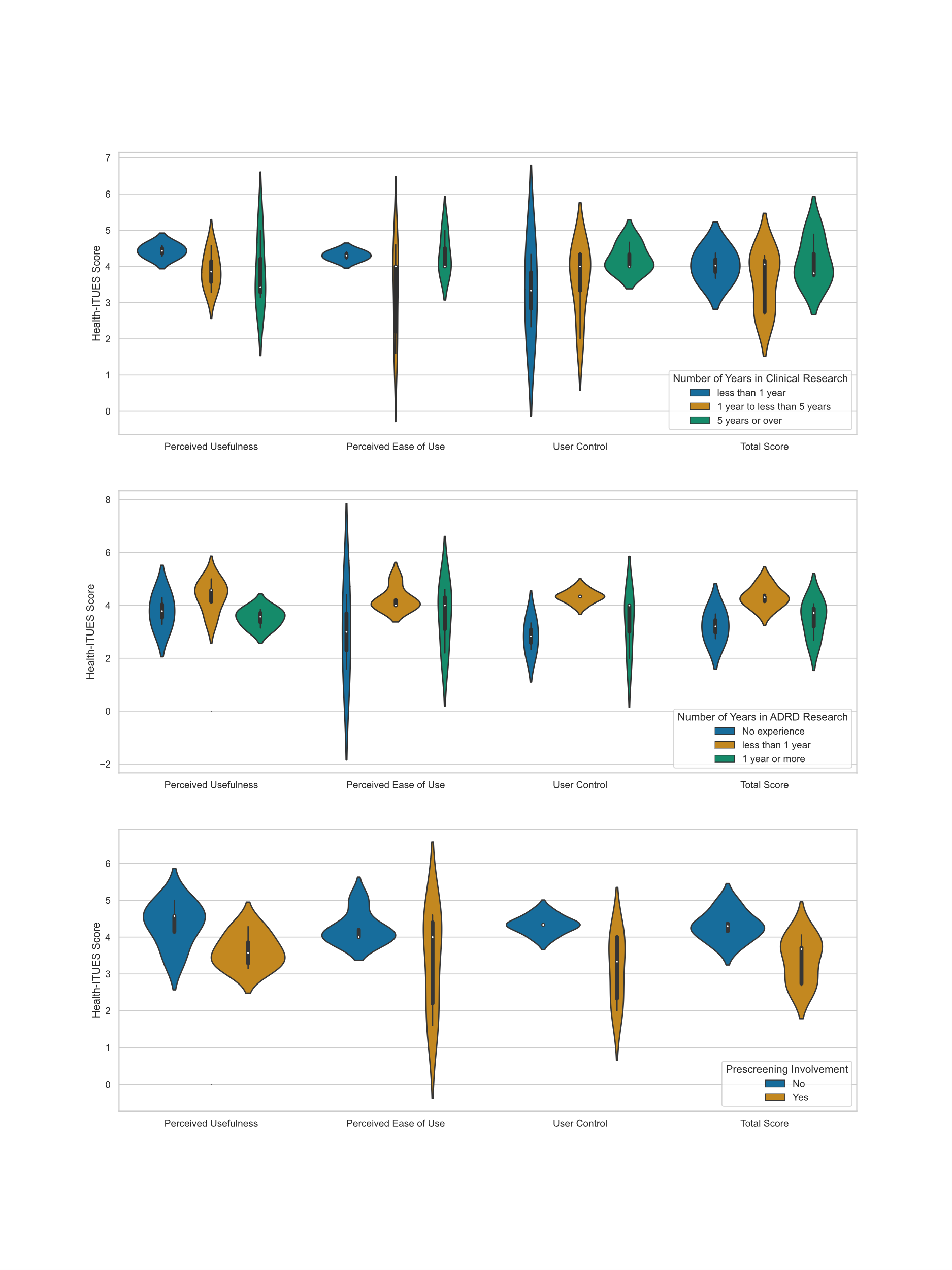

Supplement: ocac051_Supplementary_Data [file ocac051_supplementary_data.docx]
